# Application of ensemble model in capacity prediction of the CCFST columns under axial and eccentric loading

**DOI:** 10.1038/s41598-023-36576-5

**Published:** 2023-06-10

**Authors:** Jing Wang, Ruichen Lu, Ming Cheng

**Affiliations:** 1 College of Civil Engineering, Zhengzhou University of Industrial Technology, Zhengzhou, 451150 China; 2 China Construction Fifth Engineering Division Corp., Ltd., Changsha, 410000 China

**Keywords:** Composites, Mechanical properties

## Abstract

Understanding the load-carrying capacity of circular concrete-filled steel tube (CCFST) columns is crucial for designing CCFST structures. However, traditional empirical formulas often yield inconsistent results for the same scenario, causing confusion for decision makers. Additionally, simple regression analysis is unable to accurately predict the complex mapping relationship between input and output variables. To address these limitations, this paper proposes an ensemble model that incorporates multiple input features, such as component geometry and material properties, to predict CCFST load capacity. The model is trained and tested on two datasets comprising 1305 tests on CCFST columns under concentric loading and 499 tests under eccentric loading. The results demonstrate that the proposed ensemble model outperforms conventional support vector regression and random forest models in terms of the determination coefficient (R^2^) and error metrics (MAE, RMSE, and MAPE). Moreover, a feature analysis based on the Shapley additive interpretation (SHAP) technique indicates that column diameter is the most critical factor affecting compressive strength. Other important factors include tube thickness, yield strength of steel tube, and concrete compressive strength, all of which have a positive effect on load capacity. Conversely, an increase in column length or eccentricity leads to a decrease in load capacity. These findings can provide useful insights and guidance for the design of CCFST columns.

## Introduction

Concrete-filled steel tube (CFST) is an infill element consisting of an outer steel tube and a core of filled concrete^1^. The most commonly used CFST columns are circular concrete-filled steel tube (CCFST) and rectangular concrete-filled steel tube (RCFST) columns, a layout that optimizes the use of steel and concrete materials. It makes the complementary action between steel tubes and filled concrete more effective than conventional reinforced concrete and steel structural elements. On the one hand, the CFST system has mechanical advantages over reinforced concrete or pure steel members due to the restraining effect of the steel pipe on the filled concrete, which increases the strength and greatly improves the ductility of ordinary concrete^2–7^. On the other hand, the concrete core restrains the inward deformation of the steel tube, retarding the local buckling of the steel tube and enhancing the local stability of the steel tube, thus enhancing the overall stability of the column^8,9^. These synergistic effects lead to an increase in strength characteristics over the respective individual parts. Due to the advantages of high strength, resilience, good seismic energy absorption performance, and high fire resistance, CFST columns are widely used in high-rise buildings, bridges, and other structures^10,11^.

Compressive strength is the main mechanical property of CFST. Since the accurate design of CFST has an important influence on the stability of the structure, studying different techniques to analyze their compressive strength can provide a better understanding of their behavior. Currently, the experimental method and the finite element method are the two mainstream methods for predicting the performance of CFST components^12,13^. Although physical experiments provide valuable data and observations, the labor and material consumption of repeated tests are considerable. The finite element method can reduce the number of tests to some extent by computer simulation, but the results of finite element analysis depend largely on the skill level of the modeler due to the complex material properties, contact relations, boundary conditions, etc. Moreover, finite element methods often require high computer configurations^14^. With increasing interest and laboratory testing, some countries have established equation-based design standards based on extensive experimental results, such as ACI 318 (ACI 2014)^15^, Eurocode 4 (CEN 2004)^16^, AISC 360 (AISC 2016)^17^, and Chinese codes (GB 50936–2014 and GB/T 51446–2021)^18,19^. Design codes are currently the preferred method for predicting compressive strength due to their convenience and practicality. However, it's important to note that although many existing design standards can estimate strength, they have specific scopes of application. Additionally, different codes across countries may produce varying outputs under different code models, which can raise questions about the accuracy of the predictions and lead to poor decision-making by designers and engineers. Furthermore, the actual columns' material strength, geometry, cross-sectional length, and slenderness may exceed the applicability of these standards, potentially putting the structure at risk if they are used to calculate strength. Moreover, empirical formulas are typically explicit equations with a limited nonlinear relationship between inputs and outputs. In contrast, machine learning models can capture a more precise and complex mapping relationship between inputs and outputs in an implicit functional form.

For this reason, some intelligent methods need to be explored to achieve an accurate and fast output of prediction results. The development and application of machine learning techniques provide new insight to solve this problem^20^. It is foreseen that using machine learning to predict component performance will not only provide a reference for actual design but also save significant resources by making full use of completed experimental data and reducing the need for further testing^21,22^. Moreover, machine learning is based on patterns between large amounts of experimental data and is much less dependent on the users themselves. In recent years, many scholars have used machine learning algorithms such as artificial neural network (ANN), gene expression programming (GEP), back-propagation neural network (BPNN), fuzzy logic, etc. for the prediction of the ultimate bearing capacity of CFST based on the acquired datasets, and achieved good results^23–32^. For instance, researchers have employed a hybrid machine learning approach, combining artificial neural networks (ANN) with particle swarm optimization (PSO) algorithm, to predict the compressive strength of CCFST columns. The accuracy of this method has been demonstrated to surpass that of existing design codes and empirical formulas^33,34^. Ahmadi et al.^35^ used the ANN model to analyze the compression capacity of CCFT short columns under short-term axial loading, and the prediction results showed that the mean relative error of the proposed equation was 13.2%, indicating good accuracy. Hou et al.^36^ employed BPNN, genetic algorithm (GA)-BPNN, radial basis function neural network (RBFNN), Gaussian process regression (GPR), and multiple linear regression (MLR) models with diameter, length of the column, steel tube thickness, steel yield strength, and concrete compressive strength as input variables to develop prediction models for 2045 sets of CCFST data. The results showed that the developed GPR model reached higher accuracy and wider applicability than the existing design standards, and can reliably predict the strength of CCFST. Muhammad et al.^37^ achieved good accuracy R^2^ = 0.949 for ultimate axial capacity using the GEP model on 227 sets of CCFST columns, and the prediction accuracy was better than the design codes and formulas proposed by other scholars. To obtain models with higher prediction accuracy, Quang et al.^38^ employed a gradient tree boosting algorithm to predict the strength of the CFST column. Compared with random forest, support vector machine (SVM), decision tree, and deep learning, the model proposed achieved higher prediction accuracy.

In general, machine learning provides an innovative method for predicting the strength of CFST columns. Although some studies have been investigated with good results and progress, more work needs to be done for the two following reasons. (1) The current research is mainly focused on the compressive strength of CCFST under axial loading condition. Studies on the behavior of CCFST columns under different loading conditions are relatively few. A systematic and in-depth study of the mechanical properties of CCFST under different cross-sectional shapes and loading conditions is necessary. (2). The number and type of samples in the database have a significant impact on the applicability and accuracy of the mechanistic model. An extensive literature review can further supplement the number of test samples and the corresponding parameter ranges to build a more comprehensive test database. Additionally, the application of ensemble model in capacity prediction of the CCFST columns is relatively few.

**Figure 1:**
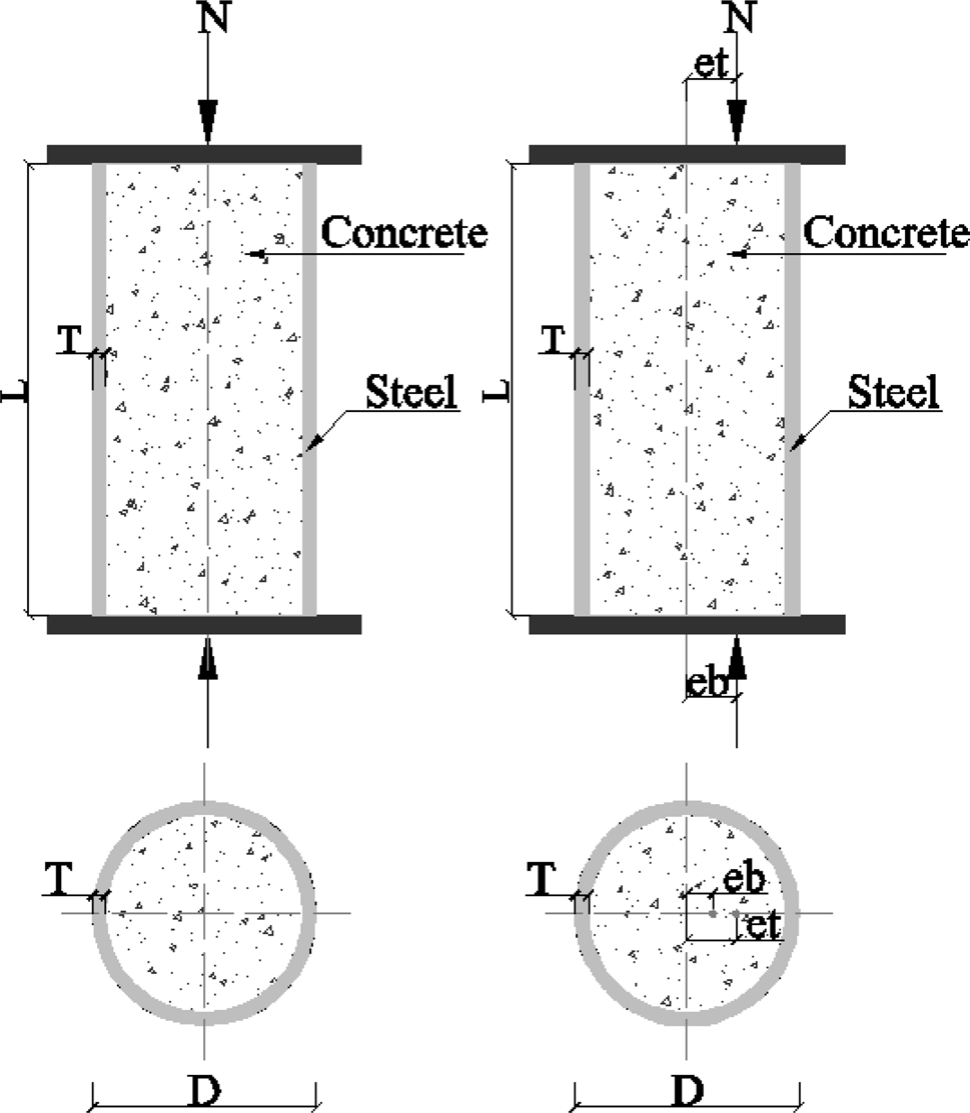
Schematic diagram of CCFST columns under axial and eccentric loading.

The main objective of this study is to develop an ensemble model that can accurately predict the compressive strength of CCFST under various loading conditions. As depicted in Fig. 1, the input parameters consist of geometric features and material properties. For CCFST, these specific input variables include diameter (*D*), the thickness of tube (*T*), length of the column (*L*), yield strength of steel tube (*f*_*y*_), concrete compressive strength (*f*_*c*_), top eccentricity (*et*), bottom eccentricity (*eb*). In light of the successful application of the Extreme Gradient Boosting model (XGBoost) in other regression problems^39^, this model was selected for prediction in this study. Additionally, two other commonly used machine learning models, support vector regression (SVR)^40^ and random forest (RF)^41^, were also employed to determine the optimal prediction model for the studied topic.

## Extreme gradient boosting model

XGBoost makes some algorithmic improvements on the basis of the GBDT gradient boosting tree, which has the advantages of being fast, effective, able to handle large-scale data, and supporting multiple languages. The basic idea is that tree by tree is added to the model, and each CRAT decision tree is added in such a way that the overall effect is improved. the objective function of XGBoost (as shown in Eq. 1) contains two parts: training error and regularization.

1 $$ obj^{t} = \sum\limits_{i = 1}^{n} {l(y_{i} ) + \Omega (f_{t} )} $$

where *l* is the loss function to measure the error between the model prediction and the true value, and *Ω* is the regularization term to measure the complexity of the model and avoid overfitting. The loss function is subjected to a second-order expansion of Taylor's formula, which leads to Eqs. (2–3).

2 $$ obj^{t} = \sum\limits_{i = 1}^{n} {l(y_{i} ,\widehat{{y_{i} }}^{t} ) + \sum\limits_{i = 1}^{t} {\Omega (f_{i} )} } = \sum\limits_{i = 1}^{n} {l(y_{i} ,\widehat{{y_{i} }}^{(t + 1)} + f_{t} (x_{i} )) + } \Omega (f_{t} ) + {\text{constant}} $$

3 $$ obj^{t} = \sum\limits_{i = 1}^{n} {[l(y_{i} ,\widehat{{y_{i} }}^{(t - 1)} ) + g_{i} f_{t} (x_{i} ) + \frac{1}{2}h_{i} f_{t}^{2} (x_{i} )]} + \Omega (f_{t} ) + {\text{constant}} $$

4 $$ g_{i} { = }\partial_{{\widehat{{y_{i} }}^{(t - 1)} }} l(y_{i} ,\widehat{{y_{i} }}^{(t - 1)} ) $$

5 $$ h_{i} = \partial_{{\widehat{{y_{i} }}^{(t - 1)} }}^{2} l(y_{i} ,\widehat{{y_{i} }}^{(t - 1)} ) $$

The basic model in this paper is a regression tree, and the complexity of the tree is jointly determined by the number of leaf nodes, the weight of each leaf node, and the penalty factor (as shown in Eq. 6).

6 $$ \Omega (f_{t} ) = \gamma T + \frac{1}{2}\lambda \sum\limits_{j = 1}^{T} {\omega_{j}^{2} } $$

where γ is the penalty coefficient, T is the number of nodes of the leaves, and w is the weight of each leaf. The objective function is transformed into Eq. (7) by ignoring the constant term and expanding the loss function and the regular term.

7 $$ obj = \sum\limits_{j = 1}^{T} {[(\sum {g_{i} } )\omega_{j} + \frac{1}{2}(\sum {h_{i} } + \lambda )\omega_{j}^{2} ]} + \gamma T $$

## Dataset description

**Figure 2:**
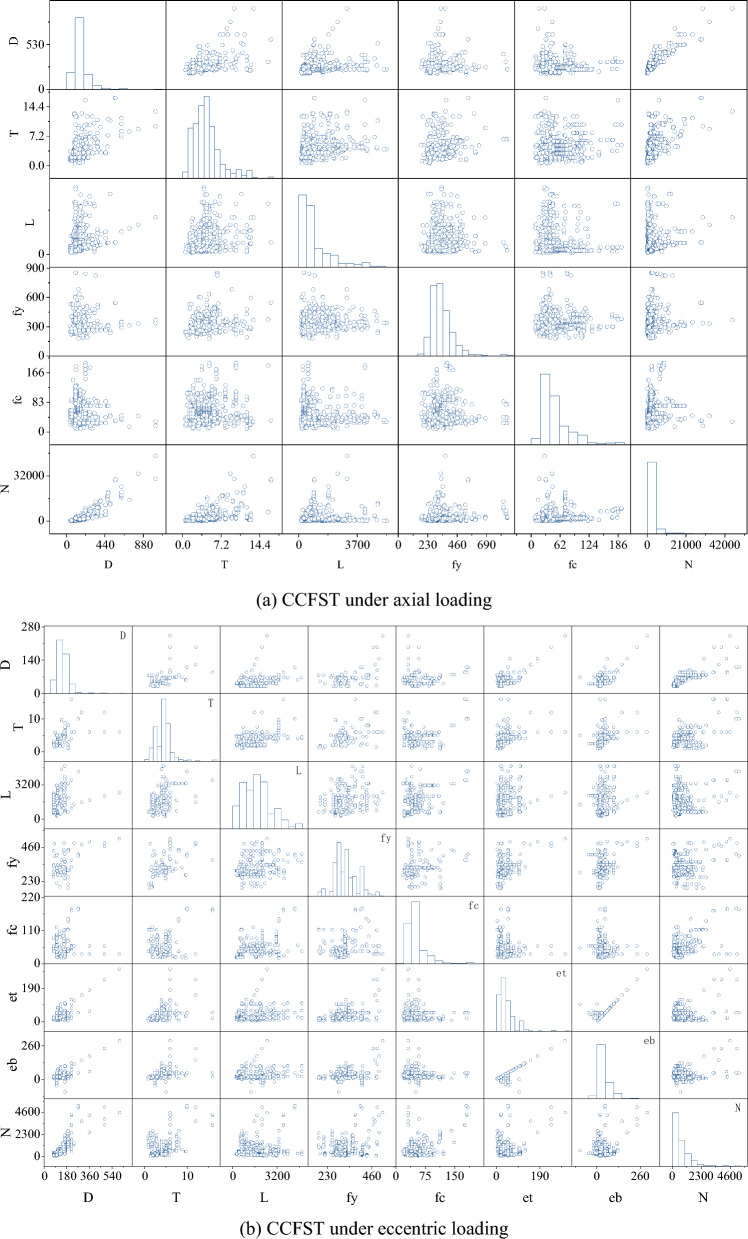
Distribution of the two datasets.

To build an accurate strength model for the CFST column, a comprehensive experimental database is required, where 1305 tests on CCFST columns under concentric loading (Dataset 1), and 499 tests on CCFST columns under eccentric loading (Dataset 2) were collected^42^. These data sets are from different laboratory experiments, although the experimental conditions may not be identical, resulting in data sets with their limitations. However, the data volume is large and the datasets are rich in sources, which are highly representative. More experimental details and descriptions of the test equipment and test conditions involved in these experimental data can be found in Reference^43^. The distributions and mathematical characteristics of these different data sets are shown in Fig. 2 and Table 1, respectively. From Fig. 2, it can be found that there is a positive correlation between the column diameter and the bearing capacity. The larger the column diameter is, the larger the compressive bearing capacity is. Similarly, the greater the thickness of the steel tube, the stronger the restraint on the internal core concrete, the more difficult it is for the concrete to deform laterally, and the bearing capacity increases. However, it can be seen that the distribution of the ultimate bearing capacity is relatively discrete, especially for CCFST under axial loading, the distribution of bearing capacity (45.2–46,000 kN) is very discrete. This poses a potential difficulty to the accurate prediction of the results.

**Table 1:** Statistical results of dataset.

Dataset	Variable	Unit	Min	Max	Median	Mean	SD	Kurtosis	Skewness
1	*D*	mm	44.45	1020	127	157.53	103.38	21.3	3.78
	*T*	mm	0.52	16.54	4	4.38	2.51	3.01	1.47
	*L*	mm	152.35	5560	660	1034.51	992.13	4.09	2.04
	*f*_*y*_	MPa	178.28	853	326	336.6	89.71	8.54	2.15
	*f*_*c*_	MPa	9.17	193.3	41.5	52.31	32.84	3.88	1.83
	*N*	kN	45.2	46,000	1308	2352.26	3957.33	40.15	5.49
2	*D*	mm	76	600	133	141.72	55.95	16.11	2.81
	*T*	mm	0.86	16	4.5	4.16	1.97	10.24	2.16
	*L*	mm	284.5	4956	1700	1757.78	1030.15	0.41	0.82
	*f*_*y*_	MPa	185.7	517	320	325.9	59.06	0.31	0.54
	*f*_*c*_	MPa	18.4	184	42.2	51.39	26.89	7.58	2.33
	*et*	mm	4	300	27.9	39.33	33.93	13.72	2.88
	*eb*	mm	-100	300	25	34.16	36.26	11.8	2.36
	*N*	kN	66.72	5288	480.2	748.94	805.62	11.78	3.06

**Figure 3:**
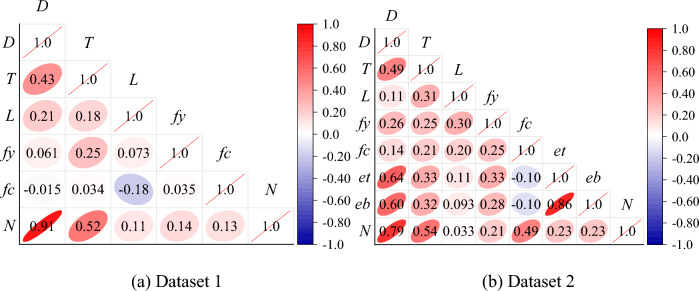
Pearson correlation coefficient of variables.

Further, the Pearson linear correlations between the input and output variables were calculated and plotted as shown in Fig. 3. As can be seen from Fig. 3, the correlation coefficient between the input and output variables in the other data sets did not exceed 0.8, except for the correlation coefficient between diameter and compressive strength in Dataset 1, which was 0.91. This implies that to achieve an accurate prediction of compressive strength, it is crucial to establish complex nonlinear correlations between multiple input variables and output compressive strength.

## Results and analysis

The collected databases were randomly divided into training datasets (80%) and test datasets (20%). It should be noted that all inputs were normalized to the range [0,1] in order to avoid scaling effects. During the training process, the grid search method was used to find the optimal hyperparameters, and the tenfold cross-validation method was employed to reduce the deviation generated by random sampling of the training set.

**Figure 4:**
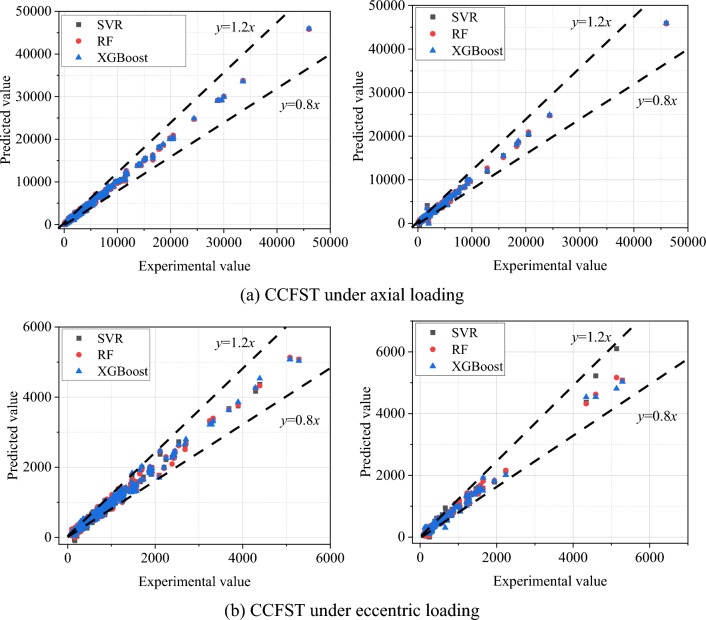
Correlation between predicted results and actual values of different models under different cases.

For comparison to assess the validity and reliability of the proposed models, random forest and the SVR model were also used for the same training and test sets. Compared to the SVR and RF models, which have fewer hyper-parameters, the tuning process of the XGBoost model is more time-consuming. However, in terms of prediction performance, the extra effort is certainly worth it. The correlation between the predicted results of the three models and the experimental values under different cases is shown in Fig. 4. It can be seen that the scatter between the predicted and actual values of the three machine learning models is mostly concentrated within ± 20% for both the training and test sets. However, the comparison of the three models is difficult to obtain from Fig. 4. For visual comparison, Table 2 lists the error metrics between the predicted results and the actual values of the different models. From Table 2, it can be found that the XGBoost model achieves higher correlation coefficients and smaller error metrics in both training and test set predictions. This is mainly because XGBoost works by combining multiple weak base models into one strong model, using a process called boosting. Boosting involves iteratively training a series of decision trees, where each new tree aims to correct the errors made by the previous trees. This iterative process continues until a stopping condition is met, resulting in an overall model that is much more accurate than any individual tree. Therefore, the XGBoost model is able to capture more complex patterns and dependencies in the data, leading to improved prediction accuracy.

**Table 2:** Evaluation indices of the prediction results of the three models.

Model	Evaluation indexes	Dataset 1		Dataset 2	
		Training	Test	Training	Test
SVR	R^2^	0.997	0.996	0.988	0.982
	MAE	136.082	162.977	63.043	88.884
	RMSE	207.608	275.212	83.602	149.849
	MAPE (%)	11.236	15.346	14.377	16.691
RF	R^2^	0.997	0.995	0.989	0.980
	MAE	151.464	174.605	57.859	74.254
	RMSE	228.251	289.435	80.311	135.276
	MAPE (%)	12.479	14.349	12.733	16.760
XGBoost	R^2^	0.997	0.996	0.989	0.989
	MAE	139.726	162.435	53.969	70.725
	RMSE	211.573	274.209	79.475	101.035
	MAPE (%)	11.433	13.923	10.496	13.805

**Figure 5:**
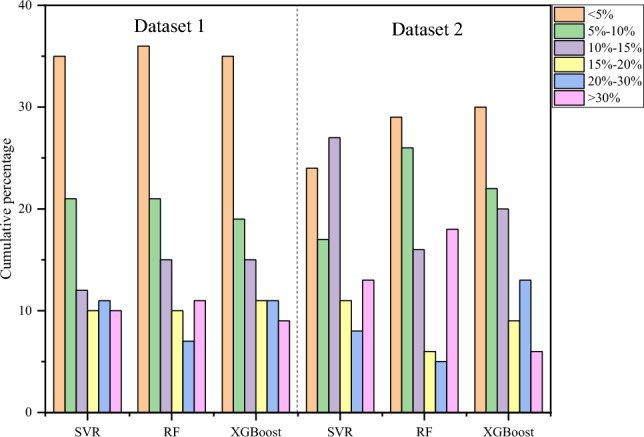
Prediction error distribution of the test set.

**Figure 6:**
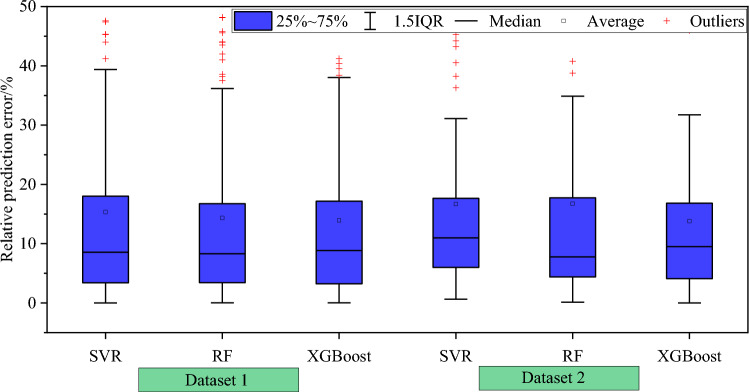
Box plot of prediction error distribution of test set.

Figure 5 shows the prediction error distribution of the models in the test set in detail. For the three machine learning models, approximately 50% of the test sets have a relative prediction error of 10% or less, and 80% of test sets have relative error distribution within 20%. Figure 6 shows the test set prediction error statistics for each model under different working conditions. For the XGBoost model, its average relative errors of prediction for the test set under the two working conditions are 13.923%, and 13.805%, respectively. The average relative errors are smaller than those of the corresponding SVR and random forest models, and the relative errors are all within 15%, which meets the requirements of engineering applications.

## Feature importance analysis

**Figure 7:**
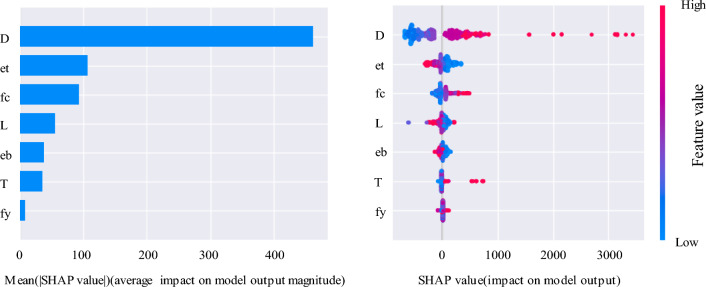
SHAP feature importance and summary plot for CCFST under eccentric loading.

The study of the importance and degree of influence of design parameters on the bearing capacity is an important guide for the design of CFST. For this reason, the Shapley additive explanation (SHAP) method is introduced in this section to analyze the influence of design parameters on the output^44,45^. As shown in Fig. 7, a high feature value greater than 0 indicates that the variable is positive for the axial compression bearing capacity, and when the high feature value is less than 0, it indicates that the corresponding variable is negative for the bearing capacity. Taking CCFST under eccentric loading as an example, the cross-sectional dimension parameter *D* is the most important design parameter under the current data set. For several other input variables, the characteristic importance of their parameters under the current data set is ranked from top to bottom. In addition, it can be concluded that all the parameters except *et*, *L*, and *eb,* are positive for the bearing capacity, and their increase will increase the bearing capacity.

## Conclusions

To further deepen the mechanical behavior of CCFST, this paper proposed an ensemble model to predict the strength of CCFST columns under axial and eccentric loading. The main conclusions are as follows.

1. The proposed ensemble model can accurately establish the complex relationships between geometry, material properties, and compressive strength for different types and different loading conditions of CCFST columns.
2. The average relative prediction errors of the proposed models for their test sets are 13.923%, and 13.805%, respectively. The average relative errors are all smaller than those of the conventional SVR and RF models, and the relative errors are all within 15%, which shows a high prediction accuracy. The proposed model can be used as an alternative to the commonly used design codes to estimate the compressive strength of CFST columns.
3. The results show that, among the input parameters considered in this study, the cross-sectional dimension (*D*) has the greatest influence on the compressive strength of CCFST columns, followed by the top eccentricity (*e*_*t*_), concrete compressive strength (*f*_*c*_), length of the column (*L*), bottom eccentricity (*e*_*b*_), and thickness of the steel tube (*T*). The yield strength of the steel tube (*f*_*y*_) has the least effect. Therefore, designers should pay close attention to the column diameter when designing CFST columns.
4. In addition, the results indicate that the top and bottom eccentricities (*e*_*t*_ and *e*_*b*_) and the length of the column (*L*) have negative effects on the compressive strength of CCFST columns, while the other geometric parameters and material properties have positive effects. This information can help designers adjust the selection of parameters in real time to achieve the best combination of design parameters for CCFST columns based on bearing capacity.

Although this research demonstrates the potential and accuracy of the ensemble learning model for predicting CCFST load carrying capacity, future research should focus on exploring the prediction effectiveness of additional machine learning models to determine the optimal prediction model. Additionally, since the dataset used in this study comes from a series of specific laboratory experiments, further verification and research are needed to assess the generalization ability of the proposed model for other similar datasets. Finally, different design parameters have varying effects on bearing capacity, and therefore it is necessary to develop an interactive graphical user interface (GUI) to assist structural designers in achieving automatic output of bearing capacity for a given input. Such a tool could aid in understanding load carrying capacity under different parameter combinations in real-time, facilitating the correction and guidance of CCFST column design.

## Data Availability

The datasets used and/or analysed during the current study available from the corresponding author on reasonable request.
